# Cryopreservation of Human Mesenchymal Stem Cells in an Allogeneic Bioscaffold based on Platelet Rich Plasma and Synovial Fluid

**DOI:** 10.1038/s41598-017-16134-6

**Published:** 2017-11-16

**Authors:** Haritz Gurruchaga, Laura Saenz del Burgo, Ane Garate, Diego Delgado, Pello Sanchez, Gorka Orive, Jesús Ciriza, Mikel Sanchez, Jose Luis Pedraz

**Affiliations:** 1 NanoBioCel Group, Laboratory of Pharmacy and Pharmaceutical Technology, Faculty of Pharmacy, University of the Basque Country, UPV/EHU, Vitoria-Gasteiz, Spain; 2 Biomedical Research Networking Center in Bioengineering, Biomaterials and Nanomedicine, CIBER-BBN, Vitoria-Gasteiz, Spain; 3 Advanced Biological Therapy Unit-UTBA, Hospital Vithas San Jose, C/Beato Tomás de Zumarraga 10, 01008 Vitoria-Gasteiz, Spain; 4 Arthroscopic Surgery Unit, Hospital Vithas San Jose, C/Beato Tomás de Zumarraga 10, 01008 Vitoria-Gasteiz, Spain

## Abstract

Transplantation of mesenchymal stem cells (MSCs) has emerged as an alternative strategy to treat knee osteoarthritis. In this context, MSCs derived from synovial fluid could provide higher chondrogenic and cartilage regeneration, presenting synovial fluid as an appropriate MSCs source. An allogeneic and biomimetic bioscaffold composed of Platelet Rich Plasma and synovial fluid that preserve and mimics the natural environment of MSCs isolated from knee has also been developed. We have optimized the cryopreservation of knee-isolated MSCs embedded within the aforementioned biomimetic scaffold, in order to create a reserve of young autologous embedded knee MSCs for future clinical applications. We have tested several cryoprotectant solutions combining dimethyl sulfoxide (DMSO), sucrose and human serum and quantifying the viability and functionality of the embedded MSCs after thawing. MSCs embedded in bioscaffolds cryopreserved with DMSO 10% or the combination of DMSO 10% and Sucrose 0,2 M displayed the best cell viabilities maintaining the multilineage differentiation potential of MSCs after thawing. In conclusion, embedded young MSCs within allogeneic biomimetic bioscaffold can be cryopreserved with the cryoprotectant solutions described in this work, allowing their future clinical use in patients with cartilage defects.

## Introduction

Osteoarthritis (OA) is a highly prevalent degenerative joint disease, that involves the cartilage and the surrounding tissue, with the pain as the clinical disease hallmark. Its incidence is increasing and prevalence grows with age, especially after the age of 50. Currently, 46 million patients suffer OA in the developed countries and this pathology might reach 70 millions by 2030^1^. In the treatment of knee OA, the implantation of autologous mesenchymal stem cells (MSCs) has emerged as an alternative to conventional therapies. Nowadays, MSCs from bone marrow are being used in the knee OA for cartilage repair, showing good safety profiles and similar effectivity than chondrocytes in the improvement of patients’ symptomatology, without major adverse effects^2–4^. However, chondrogenically induced bone marrow MSCs have the inherent risk of forming defective tissues, such as transient fibrocartilaginous tissue, calcifying cartilage and subchondral bone overgrowth^5^. Subsequently, other MSC types are actively investigated^6^. Interestingly, MSCs derived from the synovial joint tissues, such as synovial fluid (SF), synovial membrane and articular cartilage, have been proposed as alternatives due to their higher chondrogenic capacity and cartilage regeneration than bone marrow MSCs^7,8^. For example, magnetic resonance imaging, qualitative histology and Lysholm scores results from a 3-year follow-up clinical study, showed the improvement in patients with symptomatic single cartilage lesion of the femoral condyle and transplanted with MSCs derived from synovial membrane^9^. Because MSCs from the SF have similar gene expression and surface antigens profiles to MSCs from synovial membrane, with the advantage that are easier to obtain^10^, MSCs from SF might result more appropriate in the treatment of cartilage tissue.

SF is a viscous liquid composed of lubricin, hyaluronan (HA), growth factors and cytokines, mainly derivated from plasma and secretions of synoviocytes and chondrocytes^11^. Moreover, SF sometimes contains a minor presence of cells, such as MSCs, whose origin is still debated between the subchondral bone, the synovial membrane and the breakdown zone of the articular cartilage^12^. However, the migration of MSCs to the SF is enhanced while SF volume is increased, when the articular cartilage, synovial membrane, subchondral bone or the knee joint are affected, with inflammation and aggression of the intra-articular tissues^13,14^. SF is routinely extracted without harming other tissues when inflammation occurs, providing large quantities of SF from each patient. Therefore, as SF volume and MSCs number are incremented in patients suffering OA, SF could be a viable and adequate MSCs source from these patients, for their future use in the treatment of the disease.

We have developed an allogeneic and biomimetic scaffold, composed of SF and blood plasma enriched with platelets, hereafter called Platelet Rich Plasma (PRP). The mixture of PRP and SF permits the formation of an autologous bioscaffold (PRP-SF) due to the synthesis of a fibrin structure after plasma activation^15^. Our group have optimised a PRP-SF bioscaffold with an appropriate structure that shows high viabilities of embedded MSCs extracted from SF^16^.This bioscaffold can be formed during SF extraction, allowing a short preservation of embedded MSCs without the need of cell attachment and culture, and therefore, simplying the labor of the clinician in terms of time and cost^17^. Moreover, our easy and economical PRP-SF bioscaffold provides other advantages. On one hand, its size can be modulated by modifing the volume of SF and on the other hand, PRP-SF bioscaffold provides a closer environment to MSCs since it contains hyaluronic acid, growth factors and cytokines among others. Therefore, PRP-SF bioscaffold with embedded MSCs represent an alternative to the standard procedure of isolation and preservation of MSCs from SF.

Patients with OA or other cartilage defects, usually need to be treated several times during their lifes. However, while the age of the patient increases, the number, the growth potential and the replicative capacity of the MSCs from the patient decrease^18,19^. This reduction in the number and replicative potential is also reflected in the embedded MSCs within PRP-SF bioscaffold obtained from the patients. Therefore, the long time preservation of MSCs from SF or embedded MSCs within PRP-SF bioscaffold would be extremely convenient for their future clinical translation. However, to the best of our knowledge, PRP-SF has not been cryopreserved yet.

The cryopreservation of cell lines is extensively and successfully used in cell culture laboratoriesn but the cryopreservation of embedded primary cells within complex tissues or structures is still challenging^20–23^. The size of the structures represents an obstacle for the penetration of cryoprotectants (CPAs), provoking different exposition of the embedded cells to the CPA depending on the cell location in the structure, and therefore, leading to different cell viabilities throughout the structure^24,25^. Currently, two procedures are used for cryopreservation of embedded cells in 3D scaffolds: slow freezing^26^ and vitrification^21^. Vitrification has theoretical advantages over slow freezing as no ice is created in the process. However, the high CPAs concentrations required to achieve the vitreous state are toxic to cells^27^. Slow freezing is a simpler procedure with lower CPAs concentrations than vitrification^28^. Moreover, slow freezing do not need advanced equipment and can be worldwide applied in any laboratory.

In this manuscript, we have determined that DMSO 10% or the combination of DMSO 10% and sucrose 0,2 M are required for the slow freezing cryopreservation of embedded MSCs within PRP-SF bioscaffolds, allowing to move this biosystem from the bench to the clinic.

## Results and Discussion

### Isolated knee MSCs characterization

**Figure 1:**
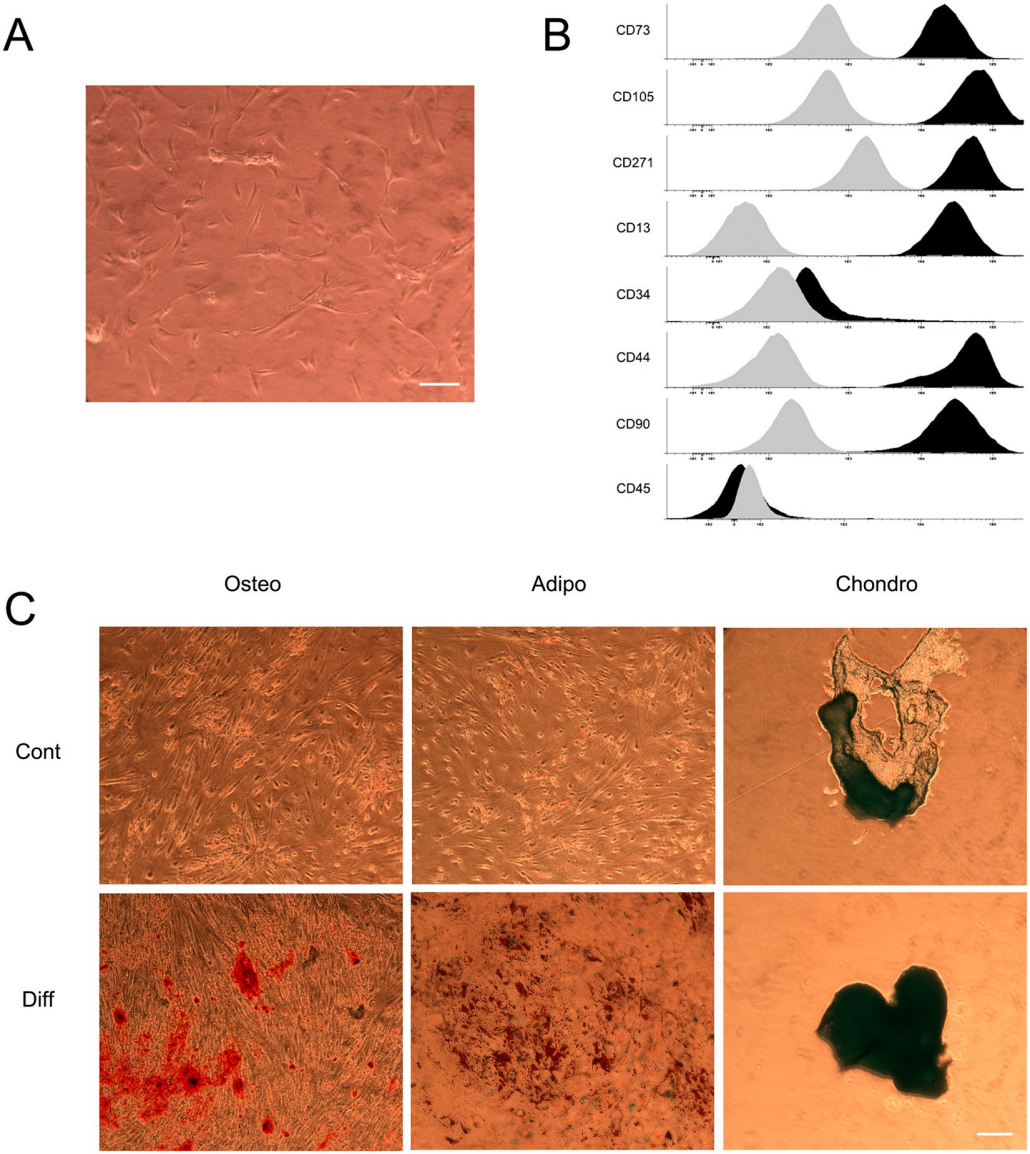
Characterization of human MSCs extracted from Synovial Fluid. (**A**) Micrograph of adhered MSCs. (**B**) Phenotypic characterization by flow cytometry of the following markers: CD73, CD105, CD90, CD34, CD45,CD271,CD13 and CD44. Grey and black histograms display minus one control and sample respectively. (**C**) Microscopic images of 3 weeks-differentiated MSCs into osteocytes (Osteo), adipocytes (Adipo) and chondrocytes (Chondro). Note: First row: undifferentiated MSCs (contr), second row: differentiated MSCs (diff). Scale bar: 100 µm.

SF is an appropriate source of MSCs because of its direct application on OA. However, SF provides lower cell number than other traditional sources, such as bone marrow^29^. Therefore, a bioscaffold that preserves the environment of the isolated MSCs is very valuable. For experimental purposes, the low number of MSCs in just 450 µL of SF is not enough for analysis, and the addition of previously isolated MSCs at higher amount in the range of at least 5 × 10^4^ cells helps to overcome this drawback. Moreover, the addition of external MSCs to the bioscaffold standardise the cell quantity in each construct. With this purpose in mind, MSCs were isolated from a donor with patellar chondropathy. Cells were cultured into a culture flask where they adhered to the bottom showing a fibroblast-like morphology (Fig. 1A). Isolated MSCs phenotype was next characterized by flow cytometry. Among several markers, it is remarkable that isolated knee MSCs expressed the mesenchymal surface markers CD73, CD105 and CD90, lacking expression of CD34 and CD45, therefore meeting the criteria established by the International Society for Cellular Therapy position for MSCs (Fig. 1B) ^30^ that identify the following qualifying criteria for MSCs: 1) cells must be adhesive to plastic, 2) cells must differentiate into chondrocytes, osteocytes, and adipocytes, and 3) cells must express the surface markers CD73, CD90, CD105 (≥95% expression)with no expression of the hematopoietic markers CD34, CD45, CD14 or CD11b, CD79α or CD19 (≤2%) and absence of HLA Class II molecules. Moreover, knee MSCs expressed the surface markers CD44, CD13 and CD271 (Fig. 1B), that correspond to the receptor of hyaluronic acid, the membrane alanyl aminopeptidase and the low-affinity nerve growth factor receptor respectively, that have been described to be expressed in MSCs^31–33^.

We also tested if knee MSCs were able to differentiate into the three mesodermal lineages. The detection of calcium deposits with the Alizarin Red, the cytoplasmatic accumulation of vacuoles filled with neutral lipids by Oil Red O, and the positive Alcian blue staining for cartilage matrix confirmed the mesenchymal potential of the isolated knee MSCs (Fig. 1C). In addition, the capacity of knee isolated MSCs to form colonies for 21 days was assessed. MSCs showed values of 53 ± 5,6% CFU-F percentage indicating its clonogenicity^34^. Therefore the isolated MSCs were used in the following experiments as an external source of cells to standardize the cell quantity in each studied bioscaffold.

### Cryoprotective comparison of PRP and PRP-SF bioscaffolds

The number, growth potential and replicative capacity of isolated MSCs decrease with the age of the patient^18,19^, a reduction that is also reflected in embedded MSCs within bioscaffold obtained from the patients. Therefore, the cryopreservation of bioscaffolds results of high interest since it allows the isolation of young MSCs for their future clinical applications.

PRP-SF bioscaffolds are composed of the mixture of PRP and SF. PRP contains fibrin, a cytocompatible structure that promotes cell proliferation due to the high diversity of binding sites that contains^35^, while SF contains HA, a glycosaminoglycan that facilitates the MSCs migration in the extracellular matrix through the interaction between HA and CD44 receptors^36^. Protective effects of HA have been described in the cryopreservation of human fibrotic monolayers^37,38^. Taking into account the additional protective properties of HA, we first aimed to determine if PRP-SF bioscaffolds, formed by PRP and SF mixture, provide a better environment than bioscaffold formed exclusively of fibrin (PRP bioscaffold), maintaining higher MSCs viabilities after cryopreservation following a conventional slow freezing protocol. Furthermore, we studied if both bioscaffolds required the presence of CPA for their cryopreservation by adding DMSO, the most common CPA in clinic and labs, and compared viabilities with non-cryopreserved bioscaffolds.

**Figure 2:**
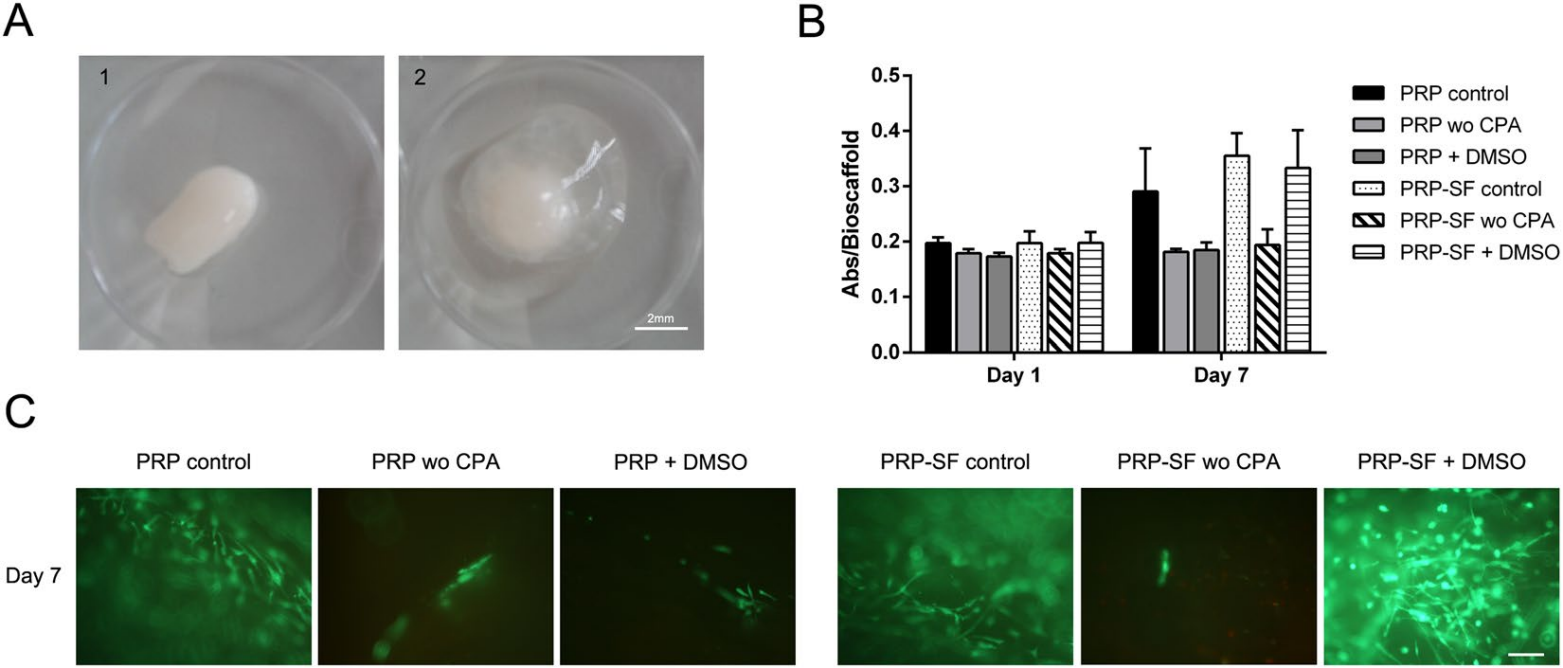
Cryoprotective comparison of PRP and PRP-SF bioscaffolds. (**A**) Macroscopic images of 1 day cultured PRP (1) and PRP-SF (2) bioscaffolds. (**B**) Viable cell number in proliferation of embedded MSCs within PRP and PRP-SF bioscaffolds 1 and 7 days after thawing. (**C**) Micrographs of calcein/ethidium stained MSCs within PRP and PRP-SF bioscaffolds 7 days after thawing. Note: PRP control: non-cryopreserved PRP bioscaffold; PRP wo CPA: PRP bioscaffold cryopreserved without CPA and additives; PRP DMSO: PRP bioscaffold cryopreserved DMSO 10%: PRP-SF control: non-cryopreserved PRP-SF bioscaffold; PRP-SF wo CPA: PRP-SF bioscaffold cryopreserved without CPA and additives; PRP-SF DMSO: PRP-SF bioscaffold cryopreserved DMSO 10%. Scale bar: 100 µm.

Once the MSC derived from the SF were characterized, PRP and PRP-SF bioscaffolds containing MSCs were tested, analyzing their capacity to maintain MSCs viability after cryopreservation and thawing (Supplemental Fig. 1). Both bioscaffolds showed different macroscopic appearance the next day after formation. PRP bisocaffold resulted in a compact solid hydrogel structure (Fig. 2A1) while PRP-SF showed a surrounding matrix formed of non-retracted SF (Fig. 2A2), that was reduced overtime (data not shown). Next day after forming bioscaffolds, no differences were quantified between the viable cell number in proliferation of non-cryopreserved PRP and PRP-SF bioscaffolds. Similarly, no differences were detected when both bioscaffolds were cryopreserved with or without DMSO, with similar viable cell number in proliferation than non-cryopreserved bioscaffolds (Fig. 2B).

However, 7 days in culture after formation or cryopreservation of bioscaffolds, differences were detected between both bioscaffolds, although not statiscally significant. PRP bioscaffolds did not protect the viable cell number in proliferation of MSCs after cryopreservation either with or without the addition of DMSO (Fig. 2B). However, PRP-SF was able to protect the viable cell number in proliferation of embedded MSCs when DMSO was added, showing similar viable cell number between non-cryopreserved embedded MSCs and those cryopreserved with DMSO (Fig. 2B), and therefore indicating that PRP-SF results in a more convenient bioscaffold for the cryopreservation of the scarce knee MSCs number. All these results were confirmed by fluorescent microscopy micrographs after calcein/ethidium staining (Fig. 2C). However, although we hypothesised that HA could have a cryoprotective effect in the PRP-SF embedded cells, we detected a decrease in embedded MSCs viable cell number in proliferation when they were cryopreserved without DMSO. In previous studies conducted with human fibrotic monolayers and solutions at high concentration (5%) of low molecular weight HA, the cryoprotective effect of HA was related to its high hydration capacity and its cell internalization CD44 receptor-mediated^37,38^. However, HA in PRP-SF bioscaffolds comes from a natural HA source and has higher molecular weight than the described in those studies. Moreover, HA in these bioscaffolds is not free in solution. Altogether precludes HA to exert its cryoprotective effect, and therefore the addition of an external CPA is required.

The lack of protection by the cryopreserved PRP bioscaffold with DMSO 10% compared to PRP-SF bioscaffold cryopreserved with DMSO 10% at day 7, could be related to cell proliferation after bioscaffold preparation. Even the desirable properties of fibrin to promote cell proliferation, and the reduction of ice creation by DMSO in cryopreservation, it seems that HA, fibrin and DMSO combination enhances cell growth after thawing. In accordance with our results, other authors have described that the proliferation and spreading of osteosarcoma cells within fibrin-HA scaffolds was significantly higher than in single (HA or fibrin) network analogues^15^. Therefore, because PRP bioscaffolds provide lower viable cell number in proliferation in embedded MSCs than PRP-SF after cryopreservation, we performed the following experiments with the MSCs embedded in the PRP-SF bioscaffolds.

### Effects and interactions of CPAs and human serum

The cryopreservation with the conventional slow freezing protocols of tissues or structures with embedded cells is still challenging. The selection and combination of CPAs is one of the most important parameters when determining the conditions for an optimal cryopreservation of such complex structures^39,40^. In function of their nature, CPAs show different cryoprotective mechanisms. Penetrant CPAs, such as DMSO, displace the internal water from the cell, minimizing the intracellular ice crystal formation while, non-penetrant CPAs, such as sucrose, act from the outside of the cells, promoting their dehydration^23^. DMSO shows several disadvantages in the preservation process such as toxicity or loss of multipotency^41^, but its replacement is difficult since no other CPA has shown the same results maintaining embedded cell viabilities after cryopreservation^42^. However, the combination of DMSO and sucrose enhance also the post-thawing viability of human MSCs^43,44^. Fetal bovine serum (FBS) has been described as another additive included in several cryopreservation protocols with beneficial effect. FBS stabilizes cell membrane, decreases the extracellular ice formation, minimizes cell dehydration and prevents excessive concentration of solutes during the freezing/thawing process^45^. However, clinicians try to avoid FBS in the cryopreservation of MSCs to reduce the risk of xeno-derived infection^46,47^, for example, with the use of human serum (HS). With all of this in mind, we studied the effect of three additives (DMSO, sucrose and HS) in the cryopreservation of MSCs embedded within PRP-SF.

**Figure 3:**
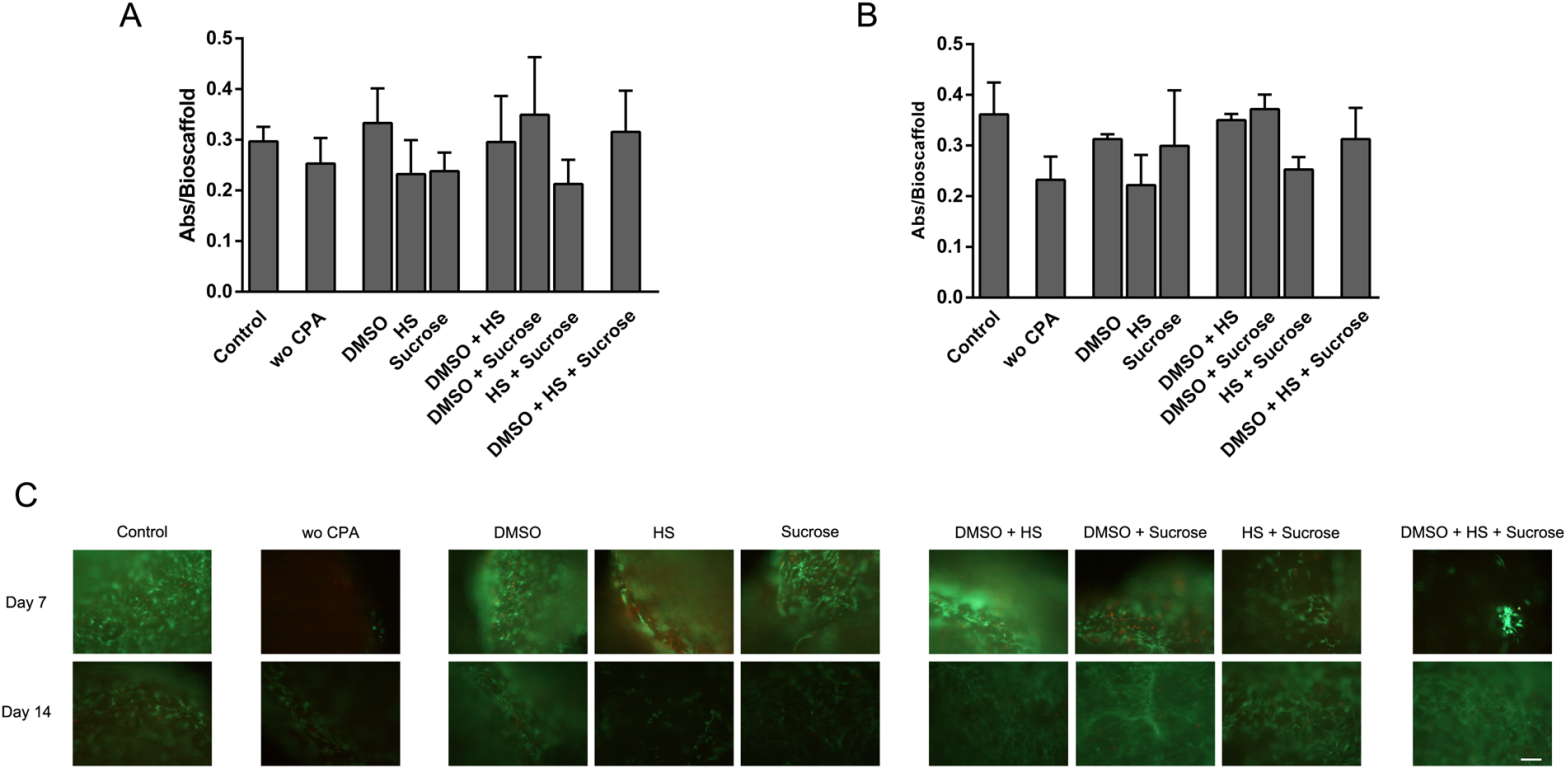
Effects and interactions of CPAs and human serum in the viable cell number in proliferation of cryopreserved embedded MSCs within PRP-SF. Viable cell number in proliferation of embedded MSCs within PRP-SF bioscaffolds 7 (**A**) and 14 (**B**) days after thawing. (**C**) Micrographs of calcein/ethidium stained MSCs within PRP-SF bioscaffolds 7 and 14 days after thawing *Note:* Control: non-cryopreserved bioscaffolds; wo CPA: without CPA and additives; DMSO: DMSO 10%; HS: Human Serum 10%; DMSO + HS: DMSO 10% + Human serum 10%; Sucrose: Sucrose 0,2 M; DMSO + Sucrose: DMSO 10% + Sucrose 0,2 M; HS + Sucrose: Human Serum 10% + Sucrose 0,2 M; DMSO + HS + Sucrose: DMSO 10% + Human Serum 10% + Sucrose 0,2 M. Scale bar: 100 µm.

We carried out a two levels three variables (DMSO, sucrose and HS) simple factorial experiment to characterize the viable cell number in proliferation of cryopreserved embedded MSCs within PRP-SF bioscaffold 7 and 14 days after thawing (timepoints when differences were expected based on previous results). When comparing bioscaffolds preserved with one additive 7 and 14 days after thawing, those preserved with DMSO 10% showed the highest viable cell number in proliferation ((Table 1 and Fig. 3A,B). This beneficial outcome was also reflected in other CPA solutions where DMSO was present, with an increasing effect on the viable cell number in proliferation at either 7 days (DMSOe = 0,0894) or 14 days (DMSOe = 0,0854) after thawing (Table 2). Fluorescent micrographs after calcein/ethidium staining confirmed the beneficial effect in the cryopreservation with CPA solutions containing DMSO of embedded MSCs within PRP-SF (Fig. 3C). We can conclude that the inclusion of a penetrant CPAs, such as DMSO, is required for the cryopreservation of MSCs embedded in PRP-SF bioscaffold since DMSO have shown the highest maintenance of embedded MSCs viable cell number in proliferation.

**Table 1:** Viable cell number in proliferation at day 7 and 14 after thawing of cryopreserved MSCs embedded within PRP-SF bioscaffolds with different CPA solutions.

Groups	DMSO (%)	HS (%)	Suc (M)	Viable cell number in proliferation			
				Day 7		Day 14	
				MEAN	SD	MEAN	SD
Control	—	—	—	0,302	0,028	0,337	0,0640
Wo CPA	0	0	0	0,253	0,045	0,232	0,043
DMSO	10	0	0	0,333	0,061	0,313	0,009
HS	0	10	0	0,232	0,061	0,222	0,061
Sucrose	0	0	0,2	0,238	0,033	0,299	0,111
DMSO + HS	10	10	0	0,296	0,079	0,351	0,012
DMSO + Sucrose	10	0	0,2	0,349	0,102	0,372	0,029
HS + Sucrose	0	10	0,2	0,213	0,043	0,253	0,025
DMSO + HS + Sucrose	10	10	0,2	0,316	0,071	0,313	0,062

Note: Control: non-cryopreserved bioscaffolds; wo CPA: without CPA and additives; DMSO: DMSO 10%; HS: Human Serum 10%; DMSO + HS: DMSO 10% + Human serum 10%; Sucrose: Sucrose 0,2 M; DMSO + Sucrose: DMSO 10% + Sucrose 0,2 M; HS +Sucrose: Human Serum 10% + Sucrose 0,2 M; DMSO + HS + Sucrose: DMSO 10% + Human Serum 10% + Sucrose 0, 2 M.

**Table 2:** Effects and interactions on viable cell number in proliferation of MSCs embedded within PRP-SF bioscaffolds at day 7 and 14 after thawing.

Effects and interactions	Viable cell number in proliferation	
	Day 7	Day 14
DMSO[e]	0,0894	0,0854
HS[e]	−0,0294	−0,0197
Sucrose[e]	0,0003	0,0298
DMSO-HS[i]	−0,062	0,0089
DMSO-Sucrose[i]	0,0178	−0,0192
HS- Sucrose[i]	−0,0002	−0,0332
DMSO-HS-Sucrose[i]	0,0019	−0,0152

Notes: [e] = effects; [i] = interations.

When HS was studied as a single preservative additive, a lower viable cell number in proliferation than embedded MSCs within PRP-SF bioscaffolds cryopreserved without CPAs was displayed (Table 1 and Fig. 3A,B). Subsequently, the HS effect on viable cell number in proliferation was negative in the factorial experiment, with values of −0,0294 at day 7 and in −0,0197 at day 14 post-thawing (Table 2), indicating that HS has not preservative properties in the cryopreservation of embedded MSCs within PRP-SF. When HS was combined with either a penetrant (DMSO) and/or a non-penetrant CPA (sucrose), only combinations in presence of DMSO displayed an increase in the viable cell number in proliferation at day 7 and 14 after thawing (Table 1 and Fig. 3A,B), indicating that the presence of HS does not improve the cryopreservative effect of the studied CPA solutions. Fluorescent micrographs after calcein/ethidium staining confirmed these results (Fig. 3C). In fact, when interactions were analyzed, only some CPA with DMSO showed positive values after thawing (Table 2). In other studies, human ovarian cortical tissues of 1–1.5 mm cryopreserved with HS and combined 1,5 M propanediol and 0,1 M sucrose showed viabilities of 65% in follicles and 75% in oocytes after thawing^48^. Although HS can provide preservative effects in other complex tissues, and it has shown better expansion and proliferation of human synovial MSCs than fetal bovine serum^49^, it seems that it is not appropriate for the cryopreservation of embedded MSCs within PRP-SF bioscaffolds.

Lastly, the addition of sucrose 0,2 M showed better viable cell number in proliferation activity than embedded MSCs within PRP-SF bioscaffolds cryopreserved without CPAs, 7 and 14 days after thawing (Table 1 and Fig. 3A,B). These results were reflected in a positive effect at both time-points: 0,003 at day 7 and in 0,0293 at day 14 (Table 2). However, embedded MSCs within PRP-SF bioscaffolds cryopreserved with sucrose 0,2 M did not reach the viable cell number in proliferation quantified in embedded MSCs within PRP-SF bioscaffolds non-cryopreserved or cryopreserved with DMSO (Fig. 3A,B). In accordance, less viable MSCs were detected in embedded MSCs within PRP-SF bioscaffolds cryopreserved with sucrose 0,2 M after calcein/ethidium staining (Fig. 3C), indicating that sucrose has protective effects in cryopreservation, but it is not enough to reach non-cryopreserved conditions. When sucrose 0,2 M was combined with DMSO, similar viable cell number in proliferation to non-cryopreserved conditions were detected (Fig. 3A–C), but this interaction did not overtake the effects obtained by only DMSO (Table 2). Altogether highlights that the studied non-penetrant CPAs have not significant cryoprotective effects, unless they are combined with a penetrant CPA such as DMSO^43,50^.

### Characterization of cryopreserved PRP-SF scaffolds

From the results described above, we conclude that the combination of DMSO and sucrose in the cryopreservation of embedded MSCs within PRP-SF bioscaffolds could provide similar results in viable cell number in proliferation than DMSO alone. Therefore, we aimed to characterized more exhaustively the effects of cryopreservation with either DMSO or DMSO combined with sucrose to define which CPA could maintain embedded MSCs in better conditions. We compared both CPA solutions with embedded MSCs within PRP-SF bioscaffolds non-cryopreserved, cryopreserved without CPA or cryopreserved with sucrose 7 days after thawing (Supplemental Fig. 2).

**Figure 4:**
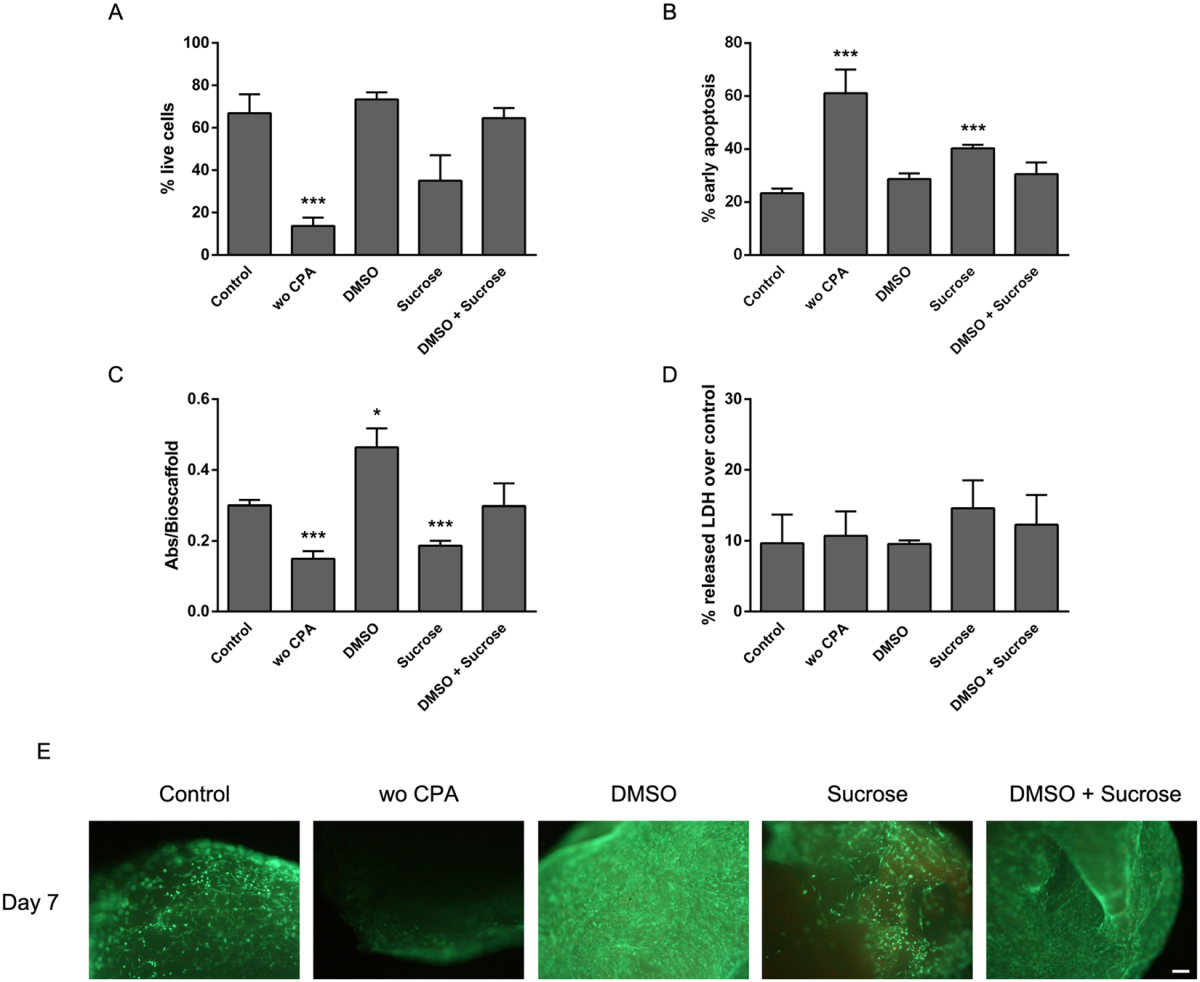
*In vitro* characterization of cryopreserved embedded MSCs within PRP-SF bioscaffolds 7 days after thawing. Quantification by flow cytometry of (**A**) live cell percentage after calcein/ethidium staining and (**B**) early apoptotic cell percentage after Annexin/PI staining. (**C**) Quantification of viable cell number in proliferation by CCK-8. (**D**) Quantification of membrane integrity by LDH release. (**E**). Micrographs of calcein/ethidium stained samples. *Note:* Control: non-cryopreserved bioscaffolds; without CPA and additives:wo CPA; DMSO: DMSO 10%; Sucrose: Sucrose 0,2 M; DMSO + Sucrose: DMSO 10% + Sucrose 0,2 M; Values represent mean ± SD. *p < 0.05, **p < 0.01 and ***p < 0.001 compared to non-cryopreserved group. Scale bar: 100 µm.

First, we determined cell viability by flow cytometry after calcein/ethidium staining. Embedded MSCs within PRP-SF bioscaffolds cryopreserved with DMSO at 10% or the combination of DMSO 10% and sucrose 0,2 M showed similar alive cells percentage (70%) than non-cryopreserved embedded MSCs within PRP-SF bioscaffolds (Fig. 4A). These viability percentages are in accordance with other studies, such as the cryopreservation of Saos-2 osteosarcoma and HaCaT embedded within poly(vinyl alcohol)-Carrageenan scaffolds^51^. Furthermore, embedded MSCs within PRP-SF bioscaffolds cryopreserved without CPAs or cryopreserved with sucrose showed lower percentage of alive cells than non-cryopreserved bioscaffolds, with significant differences (p < 0,001) in bioscaffolds cryopreserved without CPAs (Fig. 4A).

Next, we quantified the percentage of early apoptosis after Annexin-V staining. In accordance with the quantification of the percentage of alive cells, embedded MSCs within PRP-SF bioscaffolds cryopreserved with DMSO at 10% or the combination of DMSO 10% and sucrose 0,2 M showed similar early apoptotic cell percentages than non-cryopreserved embedded MSCs within PRP-SF bioscaffolds (Fig. 4B). However, compared to non-cryopreserved bioscaffolds, statistically significant (p < 0,001) higher percentage of early apoptotic cells were quantified in those samples cryopreserved without CPA or cryopreserved with sucrose, reaching values of 60% and 40% respectively. Other studies have shown the maintenance of cryoinjury in cryopreserved MSCs isolated from Wharton´s Jelly tissue with DMSO after thawing and culturing for several days, with a maximum rate of apoptotic cell percentage at 20%^52^. Although we detected higher apoptotic MSCs percentages in our cryopreserved bioscaffolds, we consider that those percentages represent the addition of two effects: cryoinjury and damaged generated by the bioscaffold breakup process required for their quantification. Nevertheless, we believe that the differences quantified among the different groups are caused by the cryoinjury phenomenon since all the samples followed the same breakup procedure.

We repeated the quantification of viable cell number in proliferation with a higher number of bioscaffolds than in the preliminary studies described above to avoid conclusions slanted by inter-variability among patients. We quantified statistically significant (p < 0,05) highest viable cell number in proliferation values in bioscaffolds cryopreserved with DMSO 10%. This effect is in accordance with those studies described by other authors, where for example, 1-month storaged MSCs shows higher proliferation rates than non-cryopreserved cells, leading to the theory of a cell selection of “stronger” cells after their storage^28,53^. Moreover, we hypothesized that changes in the PRP-SF bioscaffold network during cryopreservation with only DMSO, that maybe do not occur in the presence of sucrose, could also permit a superior spreading of the embedded cells through the bioscaffold. The combination of DMSO 10% and Sucrose 0,2 M showed similar viable cell number in proliferation than non-cryopreserved bioscaffolds, but statistically significant (p < 0,001) lower viable cell number in proliferation was quantified in samples cryopreserved without CPA or cryopreserved with sucrose (Fig. 4C).

When cell lactate dehydrogenase release was quantified, no differences among all the studied groups were identified (Fig. 4D), indicating than even when viable cell number in proliferation and apoptosis are affected, cell membrane integrity was intact. Finally, the detected differences in viable cell number in proliferation and early apoptosis results were confirmed by fluorescence microscopy after calcein/ethidium staining (Fig. 4E). Samples cryopreserved with DMSO 10% showed the highest number of alive cells, supporting the proliferation increase of MSCs previously described. Non-cryopreserved and cryopreserved with the combination of DMSO 10% and Sucrose 0,2 M samples showed high viabilities, although lower than DMSO cryopreserved samples. Sucrose 0,2 M cryopreserved bioscaffolds showed lower number of alive cells and cryopreserved bioscaffolds without CPA only showed few viable cells. Altogether, we can conclude that among the CPA studied the most appropriate for cryopreservation of embedded MSCs within PRP-SF bioscaffolds are DMSO and the combination of DMSO and sucrose, in terms of maintenance of viable cell number in proliferation activity after thawing.

### Differentiation potential of MSCs released from PRP-SF bioscaffolds

The maintenance of the multilineage capacity of the cryopreserved MSCs in the PRP-SF bioscaffolds for their posterior use in cartilage regeneration is a crucial aspect. Although several studies have confirmed that cryopreservation does not affect the differentiation capacity of MSCs^28^, the inclusion and cryopreservation of MSCs within PRP-SF could affect its potential to differentiate into osteogenic, adipogenic and chondrogenic lineages after their release from the PRP-SF bioscaffolds. Therefore, after confirming the best CPA solutions for the maintenance of viable cell number in proliferation of embedded MSCs within PRP-SF bioscaffolds, we determine if MSCs were still functional after their release from the cryopreserved PRP-SF bioscaffolds with the selected solutions.

**Figure 5:**
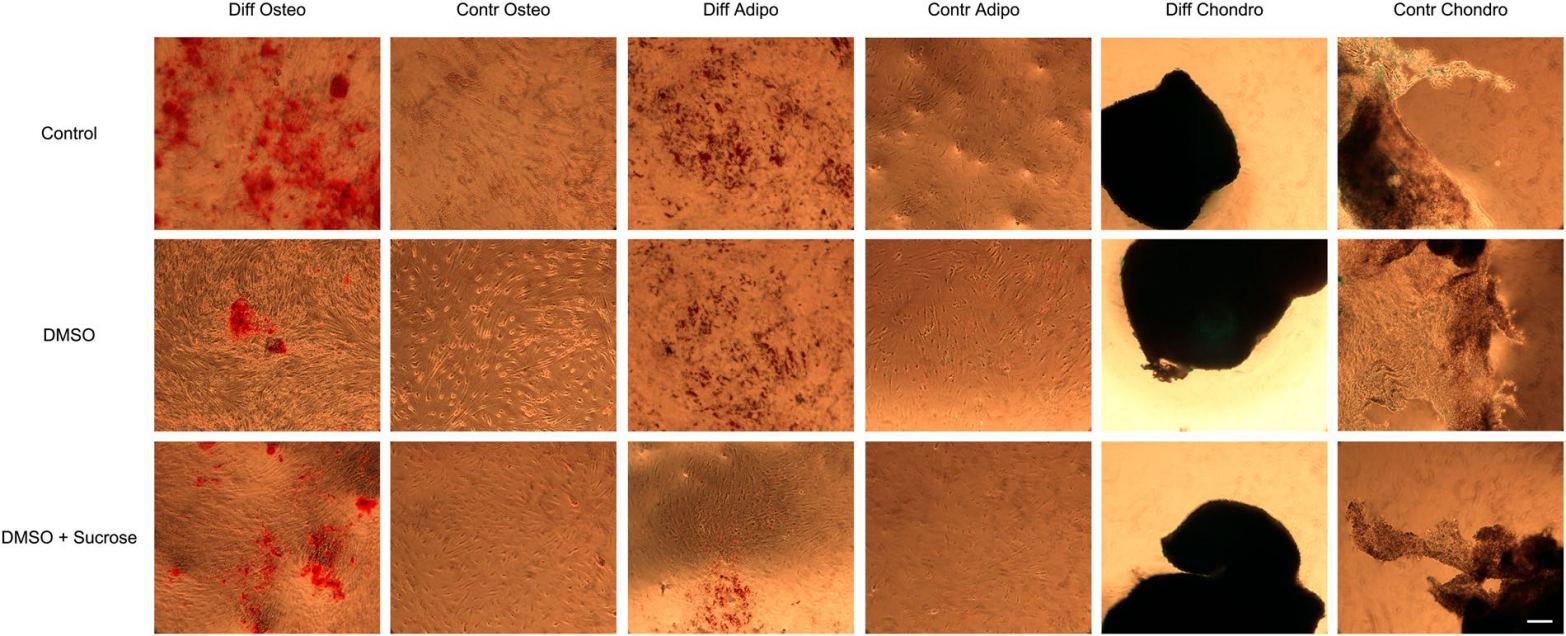
Differentiation potential of cryopreserved MSCs within PRP-SF bioscaffolds. Microscopic images of 3 weeks MSCs released from thawed PRP-SF bioscaffolds and differentiated into osteocytes, adypocites or chondrocytes. Note: Control: non cryopreserved; DMSO: DMSO 10%; Sucrose: Sucrose 0,2 M; DMSO + Sucrose: DMSO 10% + Sucrose 0,2 M. Osteo: osteogenic differentiation; Contr Osteo: non osteogenic differentiation (control); Adipo: adipogenic differentiation; Contr Osteo: non adipogenic differentiation (control); Chondro: chondrogenic differentiation; Contr Chondro: non chonrogenic differentiation (control). Scale bar: 100 µm.

After three weeks with osteogenic differentiation medium, similar calcified matrixes were observed among non-cryopreserved and cryopreserved with DMSO 10% or the combination of DMSO 10% and Sucrose 0,2 M (Fig. 5). When incubated with adipogenic differentiation medium, the presence of vacuoles were also detected in all the samples with no qualitative differences (Fig. 5). Lastly, cell pellets incubated 3 weeks with the chondrogenic differentiation medium showed positive blue staining for cartilage matrixes in all the groups (Fig. 5). With all these results it can be concluded that the cryopreservation of embedded MSCs within PRP-SF bioscaffolds does not provoke their loss of multilineage differentiation potential.

## Conclusions

PRP-SF bioscaffold provides an adequate structure and environment for the short time preservation of the scarce MSCs isolated from the synovial fluid of patients. However, healthy young MSCs should be isolated and cryopreserved within PRP-SF bioscaffolds for the future treatment of cartilage defects, since the number, growth potential and replicative capacity of MSCs decrease with age. We have demonstrated that the cryopreservation of embedded MSCs withing PRP-SF with DMSO 10% or the combination of DMSO 10% and sucrose 0,2 M provides optimal viable cell number in proliferation after thawing, while maintaining the multilineage potential differentiation properties of MSCs, importantly to chondrogenic lineage, the cell type responsible for cartilage regeneration.

## Materials and Methods

### Samples isolation and bioscaffold formation

#### MSCs Isolation from Knee Synovial Fluid (SF)

A SF sample from a 48-year-old female knee with patellar chondropathy was harvested by a syringe aspiration before intraarticular infiltration of PRP. The collected SF was diluted in phosphate buffer saline (PBS) and spin down to isolate the cellular content. Cells were seeded in a T175 flask (Corning), and cultured with Dulbecco’s Modified Eagle Medium (DMEM; Lonza) supplemented with 10% human serum (HS) in a humidified incubator at 37 °C in the presence of 5% CO_2_. Following overnight incubation, non-adherent cells were removed by replacement with fresh culture medium. Every 2–3 days, medium was removed, cells were washed with PBS (Gibco) and fresh medium was added. All the experiments, were performed with cells between 2–6 passages.

#### PRP-SF bioscaffold formation

Knee SF samples were obtained by syringe aspiration from patients before intra-articular PRP infiltration. Moreover, blood was drawn from different patients and centrifuged at 1200 g for 8 minutes at room temperature. After centrifugation, PRP was collected from the plasma fraction located above the sedimented red blood cells, but not including the buffy coat^54^. For the formation of PRP-SF bioscaffold, 450 µL of SF, 5 × 10^4^ cells (Passage 2–6) and 450 µL of PRP were mixed in a cryovial (Corning). Next, the addition of 20 µL of calcium chloride activated PRP triggering the subsequent formation of the bioscaffold. Bioscaffolds were cultured overnight in a humified incubator in the cryovials until their cryopreservation.

#### PRP bioscaffold formation

For PRP bioscaffold formation 900 µL of collected PRP and 5 × 10^4^ cells (Passage 2–6) in suspension were mixed into a cryovial, next adding 20 µL of calcium chloride to activate PRP and trigger the subsequent formation of the bioscaffold. Bioscaffolds were cultured overnight in a humified incubator in the cryovials until their cryopreservation.

The institutional review board named “Comité ético de investigación clínica (CEIC) del Hospital universitario de Araba” approved the harvest of all the samples (Code: UCA-04/EE/15/CAR), obtaining informed consents from every patient to whom biological samples were extracted. All methods were performed in accordance with the relevant guidelines and regulations.

### MSCs characterization

MSCs derived from SF were cultured and phenotyped with an 8-color direct immunofluorescence flow cytometry (Passage 2 and 6). Cell were stained with the following combination of labelled monoclonal antibodies: phycoerythrin (PE)-CD105 (clone 1G2 from Beckman Coulter), orange chrome (OC) 515-CD45 (clone GA90 from Citognos), fluorescein isothiocyanate (FITC)-CD73 (clone AD2 from BD), peridinin chlorophyll protein-cyanin 5.5 (PerCP-Cy5.5)-CD271 (clone ME204 from Biolegend), allophycocyanin (APC)-CD34 (clone 8G12 from BD), PE-cyanin 7 (PECy7)-CD13 (clone L138 from BD), Brilliant violet (BV) 421-CD90 (clone 5E10 from BD), APCH7-CD44 (clone G44-26 from BD). Negative controls of fluorescence were defined using unstained MSCs measured under the same cytometer settings as the (8-color) stained MSCs.

MSCs were also differentiated into osteoblasts, adipocytes and chondrocytes lineages to determine its multipotency (Passage 2). To induce osteogenic and adipogenic differentiation 5 × 10^5^ cells were seeded and cultured into 6-well culture plates until achievement of 70–80% confluency. Adipogenic differentiation was induced culturing with growth medium supplemented with 10% FBS (Gibco), 0.5 µM dexamethasone, 0.5 µM 3-isobutyl-1-methylxanthine, and 50 µM indomethacin. For osteogenic differentiation growth medium was supplemented with 10% FBS (Gibco), 0.05 mM L-ascorbic acid, 20 mM β-glycerophosphate and 100 nM dexamethasone. To induce chondrogenic differentiation, 4 × 10^5^ cells were cultured in 15 mL conical tubes with 0.5 mL of DMEM supplemented with 10% FBS (Gibco), 50 nM L-ascorbic acid, 6.25 µg/mL bovine insulin, and 10 ng/mL transforming growth factor-β (TGF-β) (Peprotech Inc.). Each differentiation medium was replaced every 2–3 days for 21 days. Finally, cells were fixed and stained with Alizarin Red S (osteogenic differentiation), Oil Red O (adipogenic differentiation), and Alcian Blue (chondrogenic differentiation). Reagents were purchased from Sigma-Aldrich.

The multipontecy of the embedded MSCs into PRP-SF bioscaffolds after cryopreservation was also tested (Passage 2–6). MSCs were released from PRP-SF bioscaffolds by shaking the sample with 400 µL Urokinase VEDIM 250000 UI/vial (UCB Pharma S.A) for 3 hours at 37 °C in a 5% incubator. Released cells were seeded and cultured into 6-well culture plates until achievement of 70–80% confluency, following the same aforemetioned differentiation procedures.

Fibroblast colony forming-units (CFU-F) assay was also performed to MSCs (Passage 2). Cells at passage 2 were seeded at a density of 1,000 cells/60-cm² in 6 dishes, and cultured in DMEM supplemented with 10% HS in a humidified incubator at 37 °C in the presence of 5% CO_2_. Dishes were stained after 14 days with 0.5% crystal violet and colonies were counted.

### Cryopreservation

All cryoprotectant solutions were fresh prepared by diluting the cryoprotectant (CPA) agent in maintenance medium. The following CPA solutions were prepared by the combination of dimethyl sulfoxide (DMSO) (ATCC), sucrose (Sigma-Aldrich) and human serum: DMSO 10% (DMSO), Human Serum 10% (HS), DMSO 10% + Human serum 10% (DMSO + HS), Sucrose 0,2 M (Sucrose), DMSO 10% + Sucrose 0,2 M (DMSO + Sucrose), Human Serum 10% + Sucrose 0,2 M (HS + Sucrose) and DMSO 10% + Human Serum 10% + Sucrose 0,2 M (DMSO + HS + Sucrose). One bioscaffold/cryovial was cryopreserved with 1 ml of each CPA solutions, storing at least three bioscaffold/CPA in each experiment. Cryovials without CPA or additives (wo CPA) were also stored for study. Cryovials followed the next procedure: 20 minutes on ice at 0–4 °C, overnight at −80 °C on a CoolCell (Biocision) container and final store into liquid N_2_ tanks for at least three weeks before performing any assay. Cryovials were thawed quickly at 37 °C until no ice was observed in the solution. CPA solution was slowly diluted in fresh medium to inhibit osmotic damage. Next, bioscaffolds were rinsed twice with 10 ml of maintenance medium to completely remove the CPA solution.

#### Factorial experiment

An orthogonal experimental design was followed to study the influence on cell viability of three additives in the CPA solutions: DMSO (X_1_), Sucrose (X_2_) and Human Serum (X_3_). Two levels (no presence and presence) and three variables (each additive, X_1,_ X_2_, X_3_) were evaluated in a fractional factorial experiment. The viable cell number in proliferation response at days 7 and 14, were expressed in absorbance for each experimental combination (Table 1 **)**. Three different interactions were studied in the orthogonal experimental design (X_1_X_2_, X_1_X_3_ and X_2_X_3_) and the effect of each variable and each interaction was calculated by the following equation:

$${\rm{Effect}}\,{\rm{of}}\,{\rm{Interaction}}=\frac{\sum \,{\rm{responses}}\,{\rm{with}}\,{\rm{postive}}\,{\rm{sign}}-\sum \,{\rm{responses}}\,{\rm{with}}\,{\rm{negative}}\,{\rm{sign}}}{4}$$

### Viable cell number in proliferation

The viable cell number in proliferation of MSCs within PRP and PRP-SF bioscaffolds at different timepoints after thawing was quantified by means of Cell Counter Kit 8 (CCK-8) (Sigma) assay. Briefly, complete medium was removed from bioscaffolds cultures and 1 ml of complete medium with 10% CCK-8 reagent was added incubating them for 4 hours at 37 °C in a 5% incubator. After incubation, supernatants were collected, 100 µL of each sample transferred into a 96 well plate and read on an Infinite M200 (TECAN Trading AG, Switzerland) microplate reader at 450 nm with reference wavelength at 650 nm. At least five wells were placed and three independent experiments were analysed for each condition.

### Cell viability assays with calcein/ethidum staining

The viability of MSCs within PRP and PRP-SF bioscaffolds at different time points was qualitatively assessed by fluorescence microscopy after staining with LIVE/DEAD® Viability/Cytotoxicity Kit. Bioscaffolds were rinsed three times with DPBS and then, mixed with 0.5 µM calcein AM and 0.5 µM ethidium homodimer-1 in DPBS on 24-well plates. After incubation at room temperature for 40 minutes in the dark, samples were observed under a Nikon TMS microscope with the following excitation/emission wavelengths: 495/515 nm for calcein AM and 495/635 nm ethidium homodimer. At least three independent experiments were analysed for each condition.

Cell viability was also quantified by flow cytometry in non-cryopreserved (control), wo CPA, DMSO, Sucrose and DMSO + Sucrose cryopreserved groups 7 days after thawing with the LIVE/DEAD^®^ Viability/Cytotoxicity Kit (Life Technologies). Briefly, complete medium was removed and cells were released by shaking with 400 µL Urokinase VEDIM 250000 UI/vial (UCB Pharma S.A) for 3 hours at 37 °C in a 5% incubator. Next, cells were stained with 100 nM calcein AM and 8 μM ethidium homodimer-1 solution for 20 minutes at room temperature, protected from light. Fluorescence was determined immediately with a BD FACS Calibur flow cytometerTM. Unstained samples or samples stained only with 100 nM calcein AM or 8 μM ethidium homodimer-1 were studied as controls. All the measurements were conducted in triplicates, and at least three independent experiments were analysed for each condition.

### Cell early assay apoptosis

Early apoptosis of MSCs in control, wo CPA, DMSO, Sucrose and DMSO + Sucrose cryopreserved samples was quantified 7 days after thawing with the Annexin-V-FITC Apoptosis Detection Kit (Sigma-Aldrich). Briefly, complete medium was removed and cells were released by shaking with 400 µL Urokinase VEDIM 250000 UI/ vial (UCB Pharma S.A) for 3 hours at 37 °C in a 5% incubator. Next, cells were rinsed twice with DPBS, resuspended in 10 mM HEPES/NaOH containing 0.14 M NaCl and 2.5 mM CaCl_2_ (binding buffer, pH 7.5) and stained with annexin V-FITC and propidium iodide for 10 minutes at room temperature protected from light. Fluorescence was determined immediately with a BD FACS CaliburTM flow cytometer (BD Biosciences). Unstained samples or samples stained only with annexin V-FITC or propidium iodide were analyzed as controls. All the measurements were conducted in triplicates, and at least three independent experiments were analysed for each condition.

### Cell membrane integrity assay

The release of lactic dehydrogenase (LDH) on the culture supernatants was quantified in control, wo CPAs, DMSO, Sucrose and DMSO + Sucrose cryopreserved groups 7 days after thawing as an indicator of membrane integrity using the *In Vitro* Toxicology LDH based Assay Kit (Sigma-Aldrich). Briefly, 1 ml of complete medium was incubated with PRP-SF bioscaffolds for 24 hours. After incubation, all supernatants were collected to determine the amount of released LDH. In parallel, PRP-SF bioscaffolds were also incubated for 24 h hours with 1000 µl of complete medium and lysed to determine the total LDH activity. All supernatants were subjected to enzymatic analysis based on the reduction of NAD by LDH and its further reaction with tetrazolium dye following manufacture’s recommendations. The resulting coloured compound absorbance was read out on the Infinite M200 microplate reader at a wavelength of 490 nm, with absorbance reading at 690 nm as background. All the measurements were conducted in triplicates, and at least three independent experiments were analysed for each condition.

### Statistics

Statistical analysis was performed using SPSS software, version 21.00.1. Data are expressed as means standard deviation. p < 0.05 and p < 0.001 were considered significant for comparison of groups using ANOVA, Tukey’s Post Hoc and Kruskal-Wallis H test.

## Electronic supplementary material

Supplemental figures
