# Supplementary material for: Cryopreservation of Human Mesenchymal Stem Cells in an Allogeneic Bioscaffold based on Platelet Rich Plasma and Synovial Fluid
Source: Sci Rep. 2017 Nov 16;7:15733. doi: 10.1038/s41598-017-16134-6 (PMC5691190; doi:10.1038/s41598-017-16134-6)
Supplement: Supplementary file 1 — Supplemental figures [file 41598_2017_16134_MOESM1_ESM.pdf]

TITLE: Cryopreservation of Human Mesenchymal Stem Cells in an Allogeneic Bioscaffold based on Platelet Rich Plasma and Synovial Fluid

Authors: Haritz Gurruchaga[a,b], Laura Saenz del Burgo[a,b], Ane Garate[c], Diego Delgado[c], Pello Sanchez[c], Gorka Orive[a,b], Jesús Ciriza[a,b*], Mikel Sanchez[d], Jose Luis Pedraz[a,b*]

[a] NanoBioCel Group, Laboratory of Pharmacy and Pharmaceutical Technology, Faculty of Pharmacy, University of the Basque Country, UPV/EHU, Vitoria-Gasteiz, Spain

[b] Biomedical Research Networking Center in Bioengineering, Biomaterials and Nanomedicine, CIBER-BBN, Vitoria-Gasteiz, Spain

[c] Advanced Biological Therapy Unit-UTBA, Hospital Vithas San Jose, C/Beato Tomás de Zumarraga 10, 01008 Vitoria-Gasteiz, Spain

[d] Arthroscopic Surgery Unit, Hospital Vithas San Jose, C/Beato Tomás de Zumarraga 10, 01008 Vitoria-Gasteiz, Spain

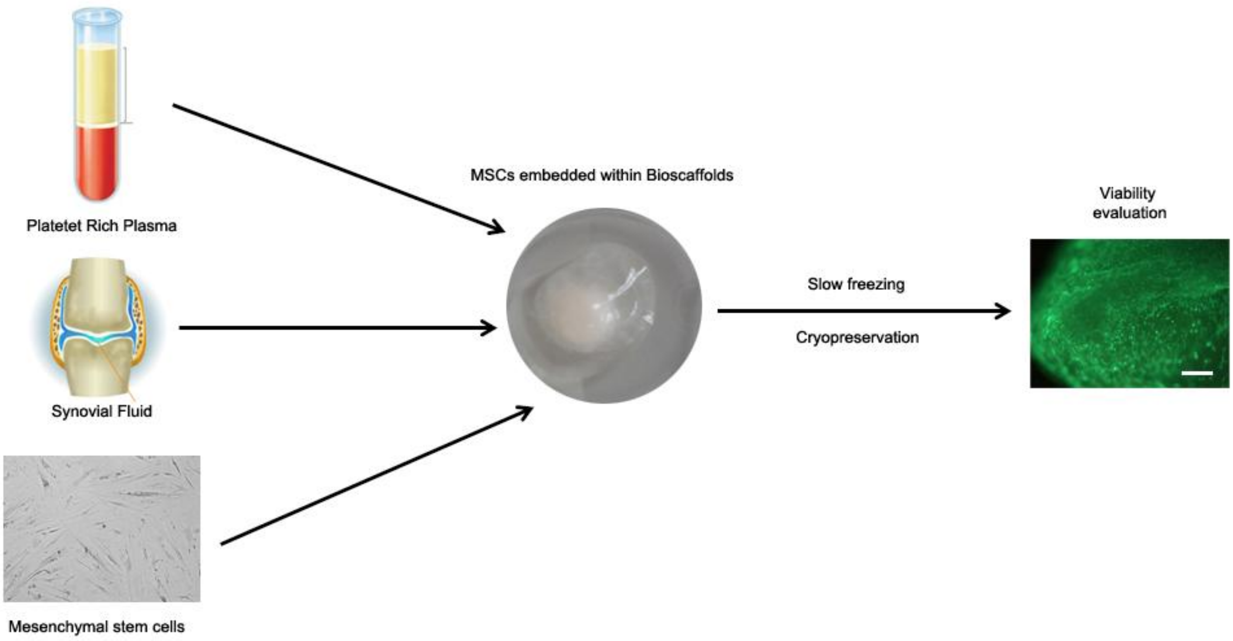

Supplemental figure 1: **Schematic illustration of the study**

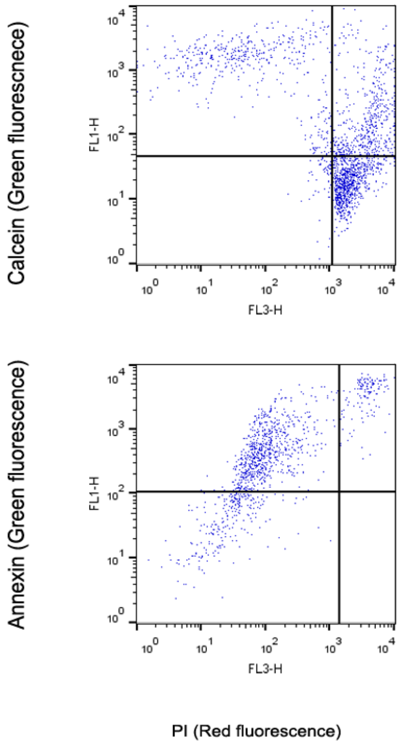

Supplemental figure 2: Representative scatter plots of the Calcein/ethidium and Annexin V staining.
